# Structure and Function of SLC4 Family ${\text{HCO}}_{3}^{-}$ Transporters

**DOI:** 10.3389/fphys.2015.00355

**Published:** 2015-12-01

**Authors:** Ying Liu, Jichun Yang, Li-Ming Chen

**Affiliations:** ^1^Key Laboratory of Molecular Biophysics of Ministry of Education, Department of Biophysics and Molecular Physiology, School of Life Science and Technology, Huazhong University of Science and TechnologyWuhan, China; ^2^Department of Physiology and Pathophysiology, School of Basic Medical Sciences, Peking University Health Science CenterBeijing, China

**Keywords:** bicarbonate transporter, solute carrier, acid-base balance, topology structure, alternative splicing, metabolic acidosis, cysteine scanning mutagenesis, N-glycosylation

## Abstract

The solute carrier SLC4 family consists of 10 members, nine of which are ${\text{HCO}}_{3}^{-}$ transporters, including three Na^+^-independent Cl^−^/${\text{HCO}}_{3}^{-}$ exchangers AE1, AE2, and AE3, five Na^+^-coupled ${\text{HCO}}_{3}^{-}$ transporters NBCe1, NBCe2, NBCn1, NBCn2, and NDCBE, as well as “AE4” whose Na^+^-dependence remains controversial. The SLC4 ${\text{HCO}}_{3}^{-}$ transporters play critical roles in pH regulation and transepithelial movement of electrolytes with a broad range of demonstrated physiological relevances. Dysfunctions of these transporters are associated with a series of human diseases. During the past decades, tremendous amount of effort has been undertaken to investigate the topological organization of the SLC4 transporters in the plasma membrane. Based upon the proposed topology models, mutational and functional studies have identified important structural elements likely involved in the ion translocation by the SLC4 transporters. In the present article, we review the advances during the past decades in understanding the structure and function of the SLC4 transporters.

## Introduction

**Figure 1:**
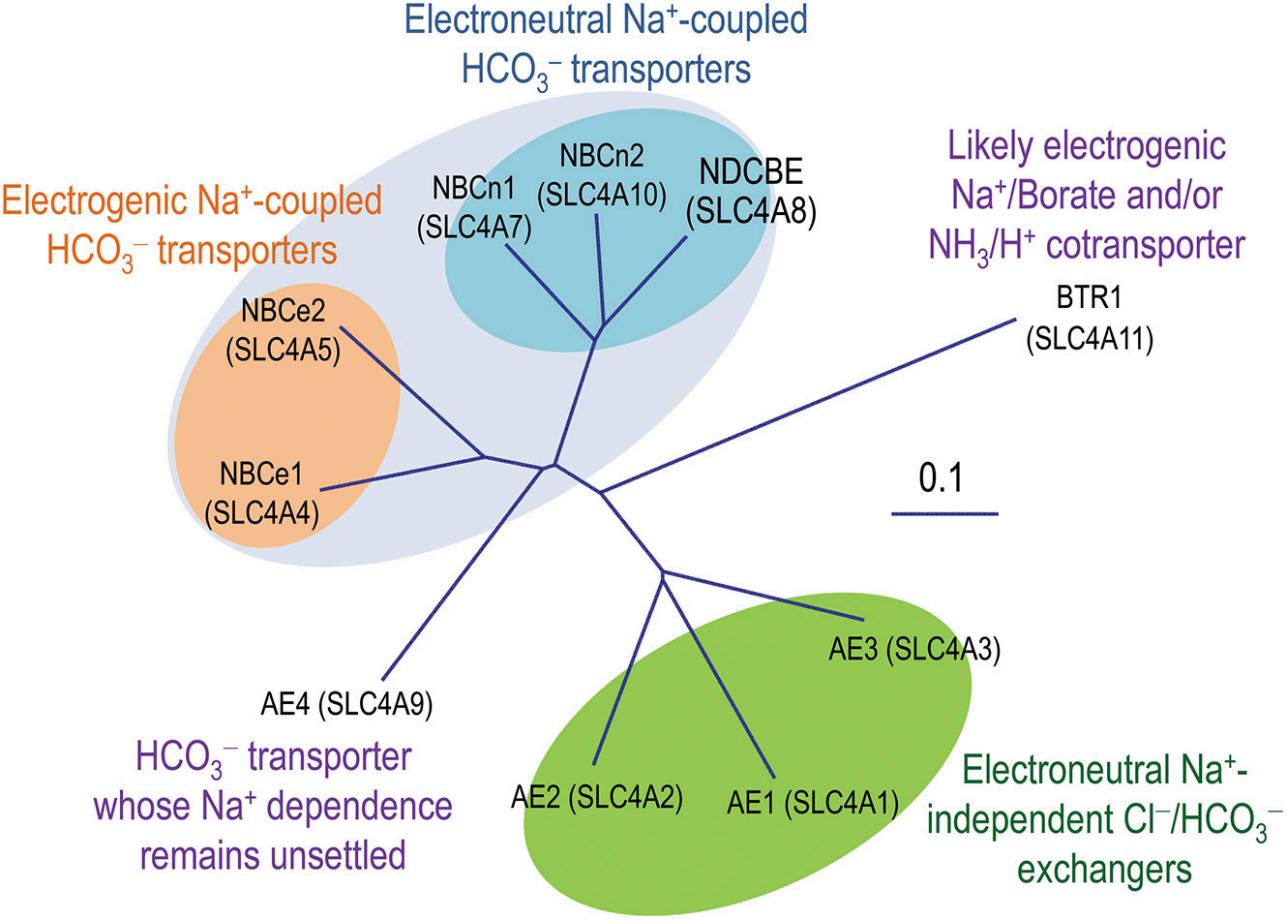
**Phylogenetic tree of SLC4 family**. A multiple sequence alignment was performed with human sequences of eAE1 (NP_000333.1), AE2a (NP_003031.3), bAE3 (NP_005061.2), AE4a (NM_031467), NBCe1-A (AAC51645.1), NBCe2-c (AAK97072.1), NBCn1-E (ACH61961), NBCn2-A (NP_071341.2), NDCBE-A (AAY79176), and BTR1 (NP_114423) with the online software Clustal Omega from the European Bioinformatics Institute. The unrooted phylogenetic tree was generated by using Treview based on the sequence alignment (Page, 1996).

It is fundamentally important to maintain acid-base homeostasis in the body. The solute carrier 4 (SLC4) family represents a major group of bicarbonate transporters. In mammals, the SLC4 family consists of 10 genes. Except for *SLC4A11* encoding BTR1 which likely mediates electrogenic Na^+^-coupled borate transport (Parker et al., 2001; Park et al., 2004) and/or electrogenic NH_3_/H^+^ cotransport (Ogando et al., 2013; Zhang et al., 2015; for review, see Patel and Parker, 2015), the other nine (*SLC4A1-5* and *SLC4A7-10*) encode ${\text{HCO}}_{3}^{-}$ transporters that are either Na^+^-independent or Na^+^-dependent (Figure 1). The Na^+^-independent members include three well characterized anion exchangers (AEs) AE1 (SLC4A1), AE2 (SLC4A2), and AE3 (SLC4A3) conducting electroneutral transmembrane exchange of Cl^−^ and ${\text{HCO}}_{3}^{-}$. The Na^+^-dependent members, commonly known as Na^+^-coupled ${\text{HCO}}_{3}^{-}$ transporters (NCBTs), include two electrogenic Na^+^/${\text{HCO}}_{3}^{-}$ cotransporters NBCe1 (SLC4A4) and NBCe2 (SLC4A5), two electroneutral Na^+^/${\text{HCO}}_{3}^{-}$ cotransporters NBCn1 (SLC4A7) and NBCn2 (SLC4A10), as well as an electroneutral Na^+^-driven Cl^−^/${\text{HCO}}_{3}^{-}$ exchanger NDCBE (SLC4A8). The Na^+^-dependence of “AE4” (SLC4A9) remains controversial. Several groups have shown evidences that the product of *SLC4A9* performs Cl^−^/${\text{HCO}}_{3}^{-}$ exchange (Tsuganezawa et al., 2001; Ko et al., 2002; Xu et al., 2003). However, Parker et al., observed in a preliminary study that human AE4 mediates Na^+^-dependent ${\text{HCO}}_{3}^{-}$ transport rather than Na^+^-independent Cl^−^/${\text{HCO}}_{3}^{-}$ exchange (Parker et al., 2002). As shown in Figure 1 and Table 1, the amino acid sequence of “AE4” is more closely related to those of the electrogenic NCBTs. Indeed, on the genomic level, the gene structure (e.g., the exon boundaries) of *SLC4A9* is more similar to those of *SLC4A4* and *SLC4A5* than to those of the three AEs (see review by Parker and Boron, 2013).

**Table 1:** **Identities of TMD among SLC4 family ${\text{HCO}}_{3}^{-}$ transporters**.

	**AE2**	**AE3**	**AE4**	**NBCe1**	**NBCe2**	**NBCn1**	**NBCn2**	**NDCBE**
AE1	73.2	67.7	42.4	44.1	42.5	42.2	42.2	42.2
AE2		71.8	44.6	45.2	42.6	42.9	42.7	43.3
AE3			44.7	44.9	43.1	44.2	43.4	43.2
AE4				62.9	59.1	53.7	53.7	53.7
NBCe1					72.3	57.4	59.8	59.8
NBCe2						52.0	56.7	55.1
NBCn1							81.5	81.5
NBCn2								84.4

*A multiple sequence alignment was performed with the TMDs of human SLC4 transporters by using the online Clustal Omega from the European Bioinformatics Institute. Note the colors denoting the groups of relatedness among the TMDs of different SLC4 family ${\text{HCO}}_{3}^{-}$ transporters. The AEs share about 70% identities with each other. The AEs share 42-45% identities with “AE4” and the NCBTs. The electrogenic NBCe1 and NBCe2 share 72% identity with each other, and they share 52-60% identities with the electroneutral NCBTs. The electroneutral NCBTs share more than 81% identities with each other. Note that, the TMD of “AE4” is most closely related to those of the electrogenic NCBTs*.

The ${\text{HCO}}_{3}^{-}$ transporters of SLC4 family are broadly expressed in the body, playing fundamental roles in intracellular and extracellular pH regulation as well as transepithelial secretion and absorption of ${\text{HCO}}_{3}^{-}$ and other electrolytes (e.g., Na^+^ and Cl^−^) in diverse tissues. The pH regulation and transepithelial ion transport mediated by these transporters are critical for a diverse range of biological processes and functions in the body, such as modulation of neuronal excitability (Hentschke et al., 2006; Jacobs et al., 2008; Sinning et al., 2011, 2015), maintenance of corneal transparency (Suzuki et al., 2012), maintenance of systemic acid-base homeostasis by the kidney (Romero and Boron, 1999; Guo et al., 2014; Kurtz, 2014), digestion-related function of the liver, the pancreas, and the duodenum (Uriarte et al., 2010; Chen et al., 2012; Park and Lee, 2012; Novak et al., 2013), regulation of vascular tone (Boedtkjer et al., 2011), development of dental enamel (Lacruz et al., 2010), bone remodeling (Riihonen et al., 2010), spermiogenesis (Medina et al., 2003; Liu et al., 2012), etc. (for review, see Alper, 2009; Boron et al., 2009; Park and Lee, 2012; Parker and Boron, 2013; Romero et al., 2013). Dysfunctions of these transporters are associated with a broad spectrum of pathological conditions, including central nervous system diseases (such as mental retardation, migraine, epilepsy, autism, drug addiction) (Sander et al., 2002; Demirci et al., 2006; Sebat et al., 2007; Gurnett et al., 2008; Krepischi et al., 2010; Suzuki et al., 2010), visual abnormalities (including cataracts, band keratopathy, glaucoma, retina degeneration) (Igarashi et al., 1999; Suzuki et al., 2010; Kao et al., 2011), cardiovascular disease (e.g., hypertension) (Barkley et al., 2004; Hunt et al., 2006; Ehret et al., 2011; Taylor et al., 2013; Wen et al., 2015a), red cell disorders (such as hemolytic anemia and Southeast Asian ovalocytosis) (Imamura et al., 1984; Jarolim et al., 1991), proximal and distal renal tubular acidosis (Bruce et al., 1997; Igarashi et al., 1999; Shayakul and Alper, 2004; Suzuki et al., 2010; Wen et al., 2015b), bone dysplasia (Lo et al., 2011), breast cancer (Ahmed et al., 2009; Antoniou et al., 2010; Mulligan et al., 2011; Lee et al., 2015), infertility (Medina et al., 2003), etc. (for review, see Alper, 2009; Parker and Boron, 2013; Romero et al., 2013; Guo et al., 2014).

Readers are referred to the latest reviews for the physiology, pathology, and the functional regulation of the SLC4 ${\text{HCO}}_{3}^{-}$ transporters (Parker and Boron, 2013; Romero et al., 2013; Thornell and Bevensee, 2015). In the present article, we will provide an extensive review on the progresses during the past decades about the structure and function of the SLC4 family ${\text{HCO}}_{3}^{-}$ transporters.

## Topological organization of SLC4 transporters

The SLC4 family transporters are N-glycosylated integral membrane proteins. The lengths and molecular weights of the polypeptides vary greatly among different members of the SLC4 family and even different variants of a same member. The lengths of the entire polypeptide of different SLC4 members vary from ~850 to 1250 amino acids (aa). The apparent molecular weights of the native SLC4 transporters, mostly with oligosaccharide moieties, range from about 90 to 200 kDa (Fairbanks et al., 1971; Tanner and Boxer, 1972; Schmitt et al., 1999; Praetorius et al., 2004a,b; Hentschke et al., 2006; Chen et al., 2007, 2008a,b; Kao et al., 2011).

**Figure 2:**
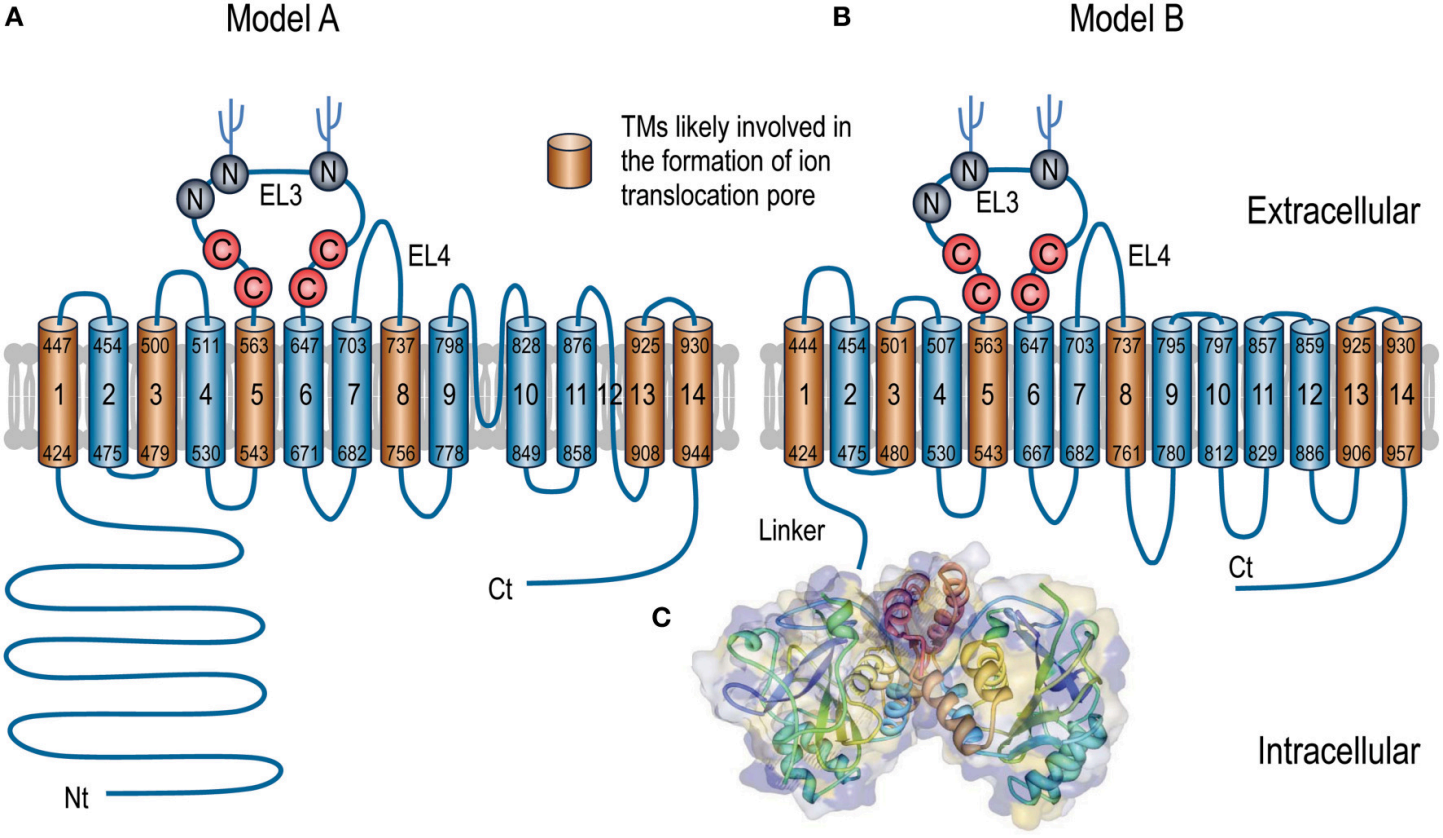
**Putative models of topological organization of SLC4 transporters (A,B) and 3D crystal structure of Nt domain of AE1 (C)**. For the convenience to compare the sequence assignments, both topological models A and B use human NBCe1-A for illustration. The numbers (according to accession #AAC51645.1) at both ends of each putative TM indicate the proposed initial and last residues of the TM in each model. Experimentally, model A is largely based on the studies with AE1 by N-glycosylation scanning mutagenesis (Popov et al., 1997, 1999) and cysteine scanning mutagenesis (Tang et al., 1998; Zhu et al., 2003). The front half of TMD (TM1-TM9) of model B is basically consistent with model A except for some minor deviations in the boundaries of TMs. The assignment of the Ct half from EL5-TM14 in model B is based upon the cysteine-scanning mutagenesis study on NBCe1 (Zhu et al., 2010a,b) and the structural modelings with AE1 (Barneaud-Rocca et al., 2013; Bonar et al., 2013). Panel C shows the 3D structure of the dimer of the Nt domain of human AE1. The model was created based on the crystal structure of the Nt of human AE1 (PDB ID #4KY9) using Protein Workshop 4.2.0 from RCSB Protein Data Bank (Moreland et al., 2005). The hydrophobicity surface (blue least, red most) was generated by using a Euclidean Distance Transform (Xu and Zhang, 2009).

As shown in Figure 2, the entire polypeptides of the SLC4 transporters contain three major domains: (1) a large intracellular amino-terminal (Nt) domain varying from ~300 to 700 aa in length; (2) a multiple-spanning transmembrane domain (TMD) of ~500 aa in length; (3) a small intracellular carboxyl-terminal (Ct) domain of ~40-130 aa in length.

The gross orientation, i.e., the intracellular localization of the Nt and Ct domains, of the polypeptides of the SLC4 family transporters has been determined largely based upon the work with AE1, the first member identified in the SLC4 family. AE1 is also known as band 3, meaning the third major band with molecular weight of about 95 kDa on sodium dodecyl sulfate polyacrylamide gel electropheresis (SDS-PAGE) of the membrane proteins from erythrocytes. AE1 is extremely abundant in erythrocytes, accounting for 25-30% of total proteins in the plasma membrane (Fairbanks et al., 1971; Tanner and Boxer, 1972). The extremely high abundance of AE1 makes the erythrocyte membrane a good system for determining the membrane orientation of AE1 in the early history of studies in the field.

### Intracellular localization of Nt domain

In the 1970s, considerable number of studies were carried out with AE1 by classical biochemical approaches to establish the intracellular localization and the gross size of the Nt domain of AE1 in erythrocytes (Drickamer, 1976, 1977, 1978; Lepke and Passow, 1976; Steck et al., 1976; Grinstein et al., 1978). In these studies, the AE1 protein in entact erythrocytes or membrane ghosts was often labeled with either radioactive reagents or specific non-radioactive chemicals and then fragmented by *in situ* protease digestion (e.g., with chymotrypsin, pronase, or trypsin) or specific chemical degradation (e.g., 2-nitro-5-thiocyanobenzoic acid, hydroxylamine, or N-bromosuccinimide). The intracellular localization of the Nt end was determined by a series of comprehensive biochemical analyses with the fragments of AE1.

Notably, extracellular treatment with chymotrypsin to entact erythrocytes splits the AE1 protein of ~95 kDa into two fragments with a molecular weight of about 65 and 30 kDa, respectively (Drickamer, 1976, 1978). The 65-kDa fragment share a same terminus with the entact 95-kDa polypeptide of AE1 (Drickamer, 1976). Moreover, as determined by carboxypeptidase digestion, this 65-kDa fragment has a different carboxyl terminus compared to the entact 95-kDa polypeptide of AE1. Therefore, it is concluded that the 65-kDa fragment represents the Nt portion of AE1 (Drickamer, 1976). Proteolytic digestion of trypsin with inside-out vesicles of erythrocytic membrane can further split the above 65-kDa fragment into a water-soluble component of ~41 kDa plus an integral component of ~17 kDa remaining in the membrane vesicle (Steck et al., 1976; Grinstein et al., 1978). Consistent with this last observation, limited trypsin digestion of the entact AE1 in human red cell ghost membranes results in an integral fragment of ~55 kDa which has an identical carboxyl terminus with the entact AE1 polypeptide as determined by carboxypeptidase digestion (Lieberman et al., 1987). Taken together, these studies demonstrate that the Nt domain resides in the cytoplasmic side as a soluble globular structure.

**Figure 3:**
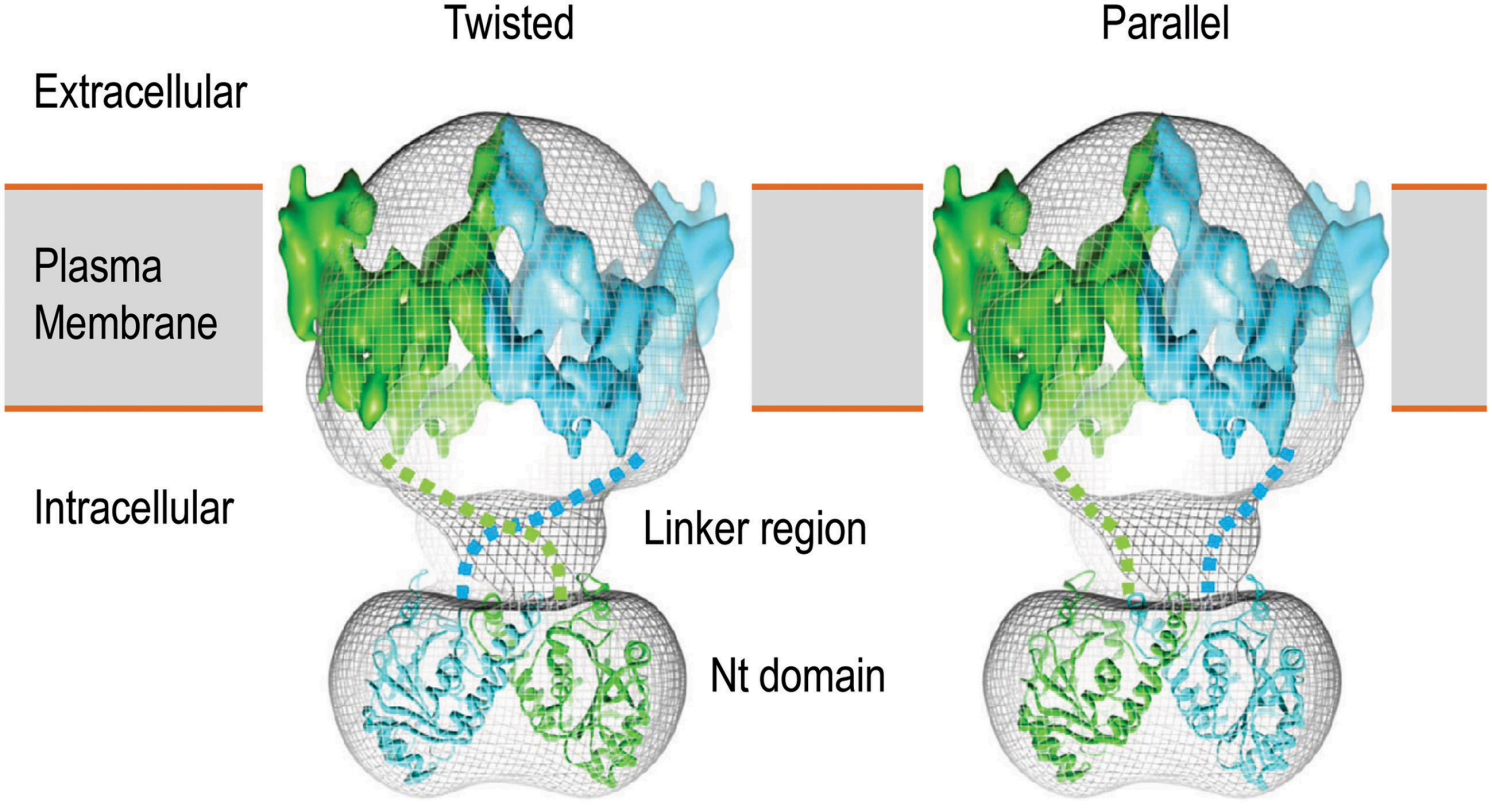
**3D structure model of human AE1 dimers obtained by cryo-EM**. Shown here are two potential organizations of the AE1 dimers: twisted (left) and parallel (right). The two molecules of AE1 are shown in different color. The figures are modified from Jiang et al. (2013).

Recombinant expression and crystallography studies on the isolated Nts of AE1 and NBCe1 have demonstrated that the Nt domains of these transporters indeed have a globular structure (Zhang et al., 2000; Gill and Boron, 2006). Single particle electron microscopy study with the full-length AE1 shows that the Nt domain is connected to the TMD by a highly flexible linker (Figure 3; see Jiang et al., 2013).

### Intracellular localization of Ct domain

The intracellular localization of the Ct domain of the SLC4 transporters was established by biochemical and immunological studies with AE1 in the late 1980s. Biochemical study showed that the carboxyl terminus of AE1 from human erythrocytes is subjected to the digestion of carboxypeptidase Y only from the cytoplasmic side of the membrane (e.g., with inside-out vesicles), but not on entact red blood cells, suggesting that the Ct domain of AE1 is located in the intracellular side (Lieberman et al., 1987).

By immunobinding assay with polyclonal antibody against the extreme Ct end of murine AE1, the Ct end of AE1 is accessible just from the cytoplasmic side of red cell membrane (Lieberman and Reithmeier, 1988). For example, inside-out vesicles of erythrocyte membrane, but not entact cells, are competitive to the binding of the antibody to the immunogen in enzyme linked immunosorbent assays (ELISA). The observation was confirmed by a study on human AE1 with monoclonal antibodies (Wainright et al., 1989). One of those monoclonal antibodies reacts with an epitope on the intracellular side of the plasma membrane of erythrocytes. Moreover, this epitope is extremely sensitive to the treatment by carboxypeptidase Y, suggesting that the epitope recognized by the monoclonal antibody is not protected by the membrane, which is consistent with the notion that the Ct end of AE1 is in the aquesous environment on the intracellular side.

### Topology structure of TMD

It has been more than four decades since the identification of AE1, the first identified member of SLC4 family, from the erythrocytes. However, the fine three-dimensional structure of the TMD of AE1 or any other SLC4 family members remains unknown. Although several 3D structures of AE1 have been reported from cryo-electron microscopy (cryo-EM) studies (Wang et al., 1994; Yamaguchi et al., 2010a,b; Jiang et al., 2013), the resolutions of these cryo-EM structures are too low to determine the topology of the TMD. Our current knowledge about the topology of the TMD of SLC4 transporters is largely derived from a series of biochemical studies on AE1 and NBCe1.

Since both the Nt and Ct domains are located in the intracellular side of the cytoplasmic membrane, it is obvious that the polypeptide of the SLC4 family transporters must span the membrane an even number of times. The cDNA of AE1 was first cloned from murine erythrocytes (Kopito and Lodish, 1985). Based on a hydropathy analysis with the primary amino-acid sequences deduced from this cDNA, the TMD of AE1 is predicted to span the cytoplasic membrane for at least 12 times (Kopito and Lodish, 1985). Since the 1980s, treandous number of undertakings have been made with AE1 and NBCe1 to determine the topology of the TMD of the SLC4 family transporters. These include biochemical studies based upon *in situ* proteolytic digestion (Hamasaki et al., 1997), cysteine scanning mutagenesis (Tang et al., 1998, 1999; Fujinaga et al., 1999; Zhu et al., 2003, 2010a,b), N-glycosylation mutagenesis (Popov et al., 1997, 1999; Cheung et al., 2005; Chen et al., 2011) as well as homology modeling (Barneaud-Rocca et al., 2013; Bonar et al., 2013).

Based upon the above studies, multiple different models have been proposed for the TMD topology of the SLC4 transporters. Generally, it has been agreed in the field that the polypeptides of the SLC4 family transporters contain 14 transmembrane segments (TMs). However, there is a major discrepancy among different models regarding the number of transmembrane alpha helices in the TMDs. Some models propose that the TMD contains 13 transmembrane helices (e.g., Zhu et al., 2003; Parker and Boron, 2013), whereas some models propose that the TMD contains 14 transmembrane helices (e.g., Popov et al., 1997; Zhu et al., 2010a,b).

Figure 2 summarizes the main features of two models emerged so far about the TMD topology of the SLC4 transporters. In model A, 13 of the 14 TMs (except for TM12) are predicted to be helical, whereas all 14 TMs in model B are predicted to be helical. The sequence assignment and orientation for TMs 1-9 and 13-14 are generally in agreement with each other in the two models, although minor differences exist regarding the precise lengths and sequence boundaries of the TMs.

Two major differences exist beween the two models for the regions between TM9 and TM13. First, the re-entrant loop RL1 (region 797-828) between TM9 and TM10 in model A is proposed to form a transmebrane helix (TM10) and an intracellular loop (IL5) in model B. Therefore, TM10 and TM11 in model A would correspond to, but be in the opposite direction with, TM11 and TM12 in model B, respectively. Second, the re-entrant loop RL2 (TM12) which is proposed to be non-helical in model A is mostly assigned to the 6th intracellular loop (IL6) in model B.

The differences in the two models reflect inconsistencies among the experimental data from different groups. Regarding “RL1” in model A, cysteine residues introduced in region 813-828 of NBCe1-A are highly sensitive to labeling by membrane permeant sulfhydryl reagent biotin maleimide (BM), but are not accessible to membrane impermeant MTS-TAMRA [2-((5(6)-tetramethylrhodamine) carboxylamino) ethyl methanethiosulfonate], consistent with the idea that this region is localized in the intracellular aquaeous environment (Zhu et al., 2010a). A consequence of this localization is that the following TM should be oriented outward, as shown in model B. Two additional lines of observations are consistent with this hypothesis: (1) Met^858^ in NBCe1-A is accessible to chemical labeling by BM and MTS-TAMRA (Zhu et al., 2010a,b), consistent with an extracellular localization of this position; (2) In AE1, N-glycosylation motif introduced at Leu^785^ (corresponding to Met^858^ in NBCe1-A) can be slightly N-glycosylated, consistent with extracellular localization of this position (Popov et al., 1999).

The putative “RL2” (the non-helical TM12) between TM11 and TM13 in model A is highly hydrophilic containing 7-8 charged residues depending on specific SLC4 members. Different studies have shown contradictory data regarding the topological localization of residues in a specific region (888-905 in NBCe1-A which corresponds to 815-829 in AE1) in RL2. There are both evidences favoring and against the intracellular localization of this region.

Evidences favoring the intracellular localization of this region include: (1) N-glycosylation motifs introduced at 820, 821, 828 of AE1 are not subjected to N-glycosylation modification in a cell-free translation system supplemented with microsomal membranes (Popov et al., 1997, 1999); (2) A monoclonal antibody directed against region 813-838 of human AE1 can react with the transporter only when the antibody is applied on the cytoplamic side of the membrane, but not when applied on intact red blood cells (Wainwright et al., 1989, 1990).

Evidences against the intracellular localization of this region include: (1) The binding of BM to cysteine residues introduced in region 815-829 of human AE1 is highly sensitive to inhibition by monobromo (trimethylammonio) bimane bromide (qBBr), which is believed to be membrane impermeant (Zhu et al., 2003); (2) Cysteine residues introduced in region 815-829 of human AE1 can be labeled by lucifer yellow iodoacetamide (LYIA), a reagent that is believed to be membrane impermeant (Zhu et al., 2003).

**Figure 4:**
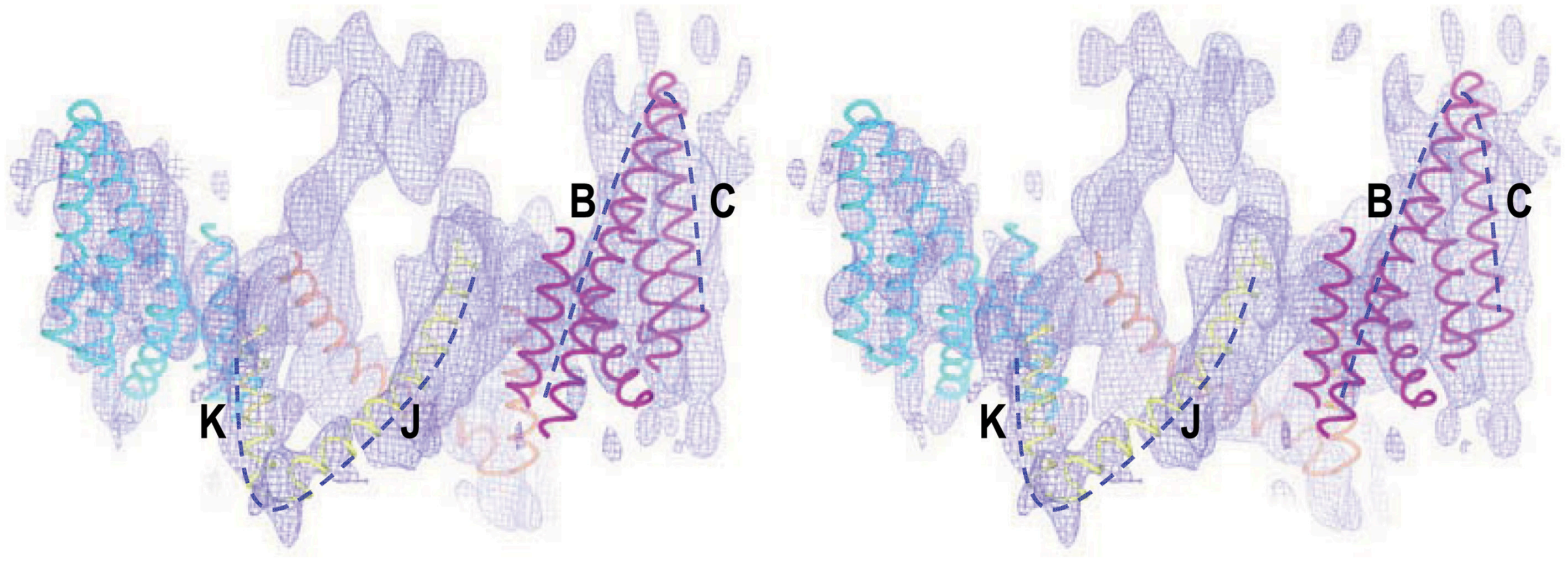
**Stereo view of cryo-EM structure of the TMD of human AE1 fitted with structural elements of chloride channel ClC**. The dotted lines show the two V-shaped structures in the TMD of AE1 that fit well with helices B + C and J + K of ClC, respectively. The figure was modified from Yamaguchi et al. (2010b) with permission.

Interestingly, a cryo-EM structure at a resolution of 7.5Å reveals that the TMD of human AE1 contains two V-shaped structures arranged in opposite direction to each other in the membrane (Figure 4; see Yamaguchi et al., 2010b). The authors therefore proposed that these two V-shaped structures represent two inverted repeats in AE1 TMD. Such inverted repeats are characteristic of a number of integral membrane transporters and channels (see review by Abramson and Wright, 2009), such as ClC chloride channel (Dutzler et al., 2002), uracil-H^+^ symporter UraA (Lu et al., 2011), Na^+^-galactose cotransporter vSGLT(Faham et al., 2008), Na^+^-leucine cotransporter LeuT (Yamashita et al., 2005), aquaporins (Murata et al., 2000; Sui et al., 2001), and glycerol facilitator GlpF (Fu et al., 2000). The TMDs of these integral membrane proteins usually contain an even number of transmembrane helices and have a pseudo two-fold symmetry (see review by Abramson and Wright, 2009). Some of these proteins (e.g., AQP1 and ClC) contain two half-helices in the membrane pointing to each other in opposite direction that play important roles in substrate binding or the formation of the ion selectivity filter (Murata et al., 2000; de Groot and Grubmuller, 2001; Dutzler et al., 2002).

The presence of two V-shaped structures (presumably two inverted repeats) in the cryo-EM structure of AE1 appears to favor topological model B which has 14 transmembrane helices (Hirai et al., 2011). Based upon the cryo-EM structure (Yamaguchi et al., 2010b), two topological models of the TMD human AE1 have been proposed by homology modelings, one with the crystal structure of UraA transporter as template (Barneaud-Rocca et al., 2013), and the other with the crystal structure of ClC as template (Bonar et al., 2013). In both models, the TMD of AE1 was predicted to contain 14 transmembrane alpha helices.

## Role of Nt domain in SLC4 transporters

**Figure 5:**
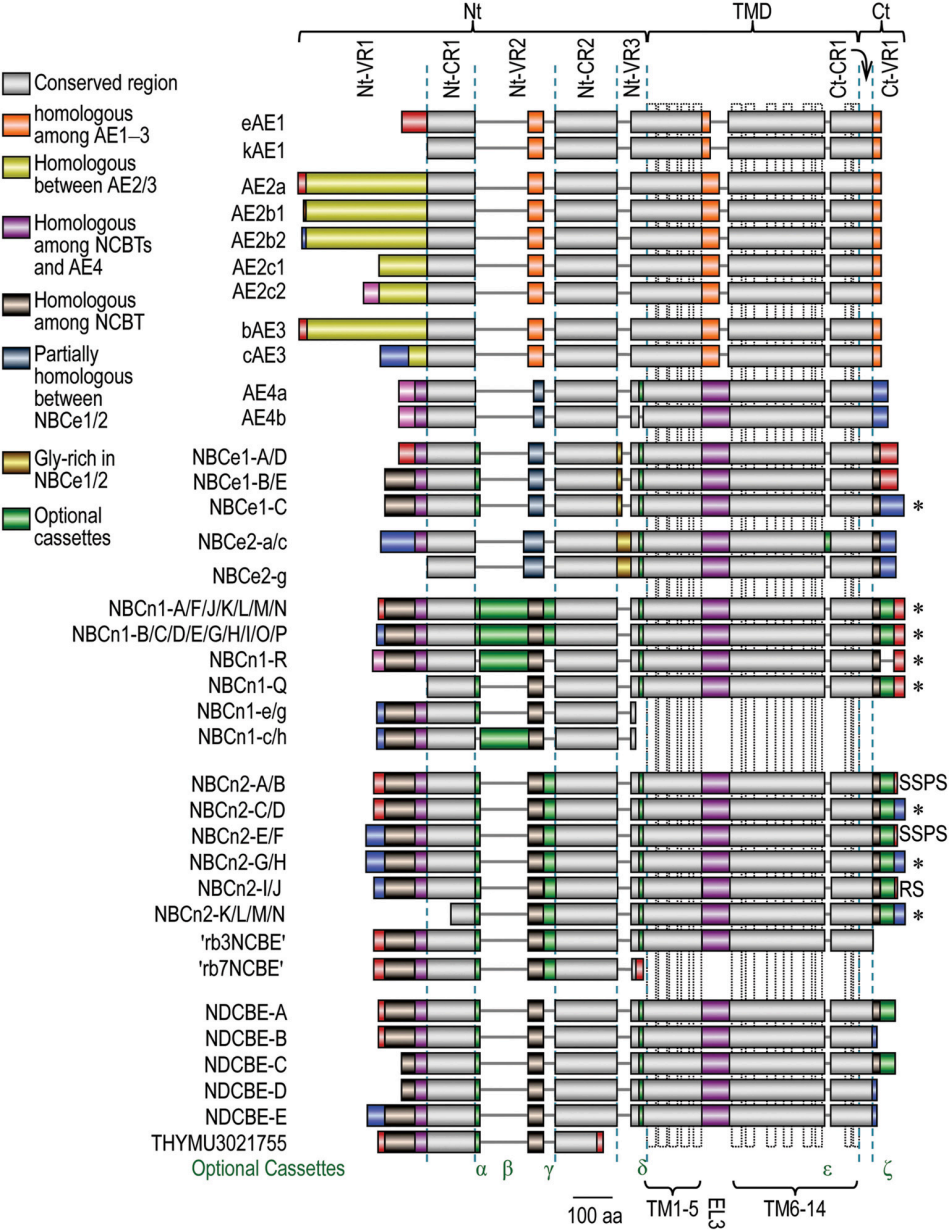
**Alignment of expression variants of SLC4 family ${\text{HCO}}_{3}^{-}$ transporters**. The diagram was based upon a sequence alignment with Clustal Omega from the European Bioinformatics Institute Details. Accession numbers for the SLC4 variants are available in Supplemental Table S1. The colors denoting homology among different SLC4 members are shown in the legend. The extreme Nt and Ct ends of different SLC4 members with identical color pattern do not denote homology unless specified elsewhere in the main text. The dotted vertical boxes in TMD indicate the putative transmembrane helices shown in model A in Figure 2. ^*^Proteins containing PDZ-binding motif at the Ct end.

The Nt domain accounts for 32-55% of the entire polypeptides of the SLC4 family transporters. As shown in Figure 5, the Nt domain can further be devided into multiple subdomains, including three variable regions Nt-VR1, Nt-VR2, and Nt-VR3 as well as two conserved regions Nt-CR1 and Nt-CR2. The conserved regions are highly homologous with a sequence identity of more than 33% among different SLC4 members, and 64-82% among different NCBTs. In contrast, the variable regions usually have little similarity among different SLC4 members and different variants of a same SLC4 member.

Crystallography studies show that the Nt domains of the SLC4 transporters exist as homodimers (Zhang et al., 2000; Gill and Boron, 2006; Shnitsar et al., 2013). In the fine 3-dimensional (3D) crystal structures of the Nt domain of AE1, the variable regions (Nt-VR1 and Nt-VR2) are largely invisible in the electron density map, suggesting that these regions are intrinsically disordered. In contrast, Nt-CR1 and Nt-CR2 are intertwined to form a compact structure with a typical “α + β” fold that have segregated alpha helices and beta strands. This compact structure represents the core structure of the Nt domain (Figure 2C). As an intracellular globular domain, the Nt is connected to the TMD by a highly flexible linker as revealed by single particle electron microscopy study (Jiang et al., 2013).

Interestingly, the Nt domains of AE1 and NBCe1 have a fold similar with the EIIA protein (http://scop.mrc-lmb.cam.ac.uk/scop/data/scop.b.e.cec.A.html; see review by Parker and Boron, 2013). The EIIA protein is a component of the bacterial nitrogen-metabolic phosphotransferase system (Deutscher et al., 2006). It has been shown that EIIA can interact with and regulate the membrane potassium transporter TrkA in *Escherichia coli* (Lee et al., 2007).

A key question is what the fundamental role of the large intracellular Nt domain is in the ion translocation process by the SLC4 transporters. Is the Nt just a regulatory domain involved in functional modulation of the transporter? Is it also a part of the ion translocation machinery (i.e., a structural component of the ion translocation pathway) that is indispensable for the transport activity of the proteins?

It is clear that the Nt domains of the SLC4 family transporters are involved in the functional regulation of these transporters in diverse manners. Firstly, the Nt domain of AE1 contains binding sites for multiple cytoskeletal proteins, such as ankyrin-1 (Bennett and Stenbuck, 1980; Grey et al., 2012), protein 4.1 (An et al., 1996), protein 4.2 (Toye et al., 2005; Bustos and Reithmeier, 2006), adducin (Anong et al., 2009), etc. These protein interactions are critical for maintaining the stability and integrity of the plasma membrane of erythrocytes (see review by Alper, 2009). At least, the interaction with protein 4.2 has stimulatory effect on the transport activity of AE1 in *Xenopus* oocytes (Toye et al., 2005). Secondly, the Nt domain of AE2 contains multiple structural elements important for the acute regulation of transport activity by changes in intracellular and extracellular pH (see discussion in “Structural variations in SLC4 transporters”). Thirdly, as will be discussed in details in section “Structural variations in SLC4,” the Nt domains of the NCBTs contain multiple optional structural elements (OSEs), some of which contain binding sites for regulatory protein partners or can modulate the intrinsic activity of the transporters.

The next question is whether the Nt domain of the SLC4 transporters is a structural component of the ion translocation pathway that is necessary for the transport activity? Evolutionally, it appears that the large Nt domain of SLC4 transporters was originated from a pre-existing gene independent of the one encoding the TMD. The SLC4-like transporters from bacteria, fungi, and plant lack the large Nt domain that is present in the mammalian homologs. However, an isolated gene homologous to the encoding sequence of the Nt domains of SLC4 transporters is not identifiable in any available genome (see review by Parker and Boron, 2013). The fact that the SLC4-like transporters from bacteria, fungi, and plant lack the large Nt domain raises a question whether the Nt domain is indispensable for the activities of the mammalian SLC4 transporters.

The answer to this question appears to be different for the AEs and NCBTs. It is now clear that the Nt domain is not necessary for the transport activity of the anion exchangers of SLC4 family (at least for AE1 and AE2). Studies have shown that the transport activity of the anion exchangers is largely retained even with the entire Nt domain removed (Grinstein et al., 1978; Stewart et al., 2001; Shnitsar et al., 2013). For example, by using inside-out vesicles, it has been shown that removal of the Nt-domain by trypsinization has no effect on the anion-exchange activity of the membrane vesicles (Grinstein et al., 1978). Moreover, human AE1 variant with the entire Nt domain truncated is well expressed on the plasma membrane and contains full transport activity in human embryonic kidney cell line HEK-293 (Shnitsar et al., 2013). Thirdly, mouse AE2 mutant with the entire Nt domain removed is almost fully active in mediating anion exchange when heterologously expressed in *Xenopus* oocytes (Stewart et al., 2001).

A series of studies have shown that the variable region Nt-VR1 is not necessary for the surface expression and transport activity of NCBTs (McAlear et al., 2006; Parker et al., 2007a; Fukuda et al., 2013; Wang et al., 2015). When heterologously expressed in *Xenopus* oocytes, NBCn1-Q, a natural variant of *Slc4a7* lacking Nt-VR1, contains minimal ${\text{HCO}}_{3}^{-}$ transport activity and typical Na^+^-conductance characteristic of NBCn1 (Wang et al., 2015). However, removal of the entire Nt domain almost completely inactivates rat NBCe1-C when heterologously expressed in *Xenopus* oocytes, although this molecular maneuver has little effect on the surface expression of NBCe1 (McAlear et al., 2006).

Based on a study with human NBCe1-A, Chang et al. proposed that the Nt domain of NBCe1, and likely other SLC4 family members, contains a substrate entry tunnel controlling the ${\text{HCO}}_{3}^{-}$ permeation of the transporter (Chang et al., 2008). By molecular modeling based on the 3D crystal structure of AE1-Nt, the authors claim that Glu^91^ and Arg^298^ in NBCe1-A Nt are located in the pocket of the hypothetical substrate entry tunnel and interact with each other by charge-charge bonding (Chang et al., 2008). These two residues are completely conserved among all SLC4 ${\text{HCO}}_{3}^{-}$ transporters. Consistent with the hypothetical interaction between Glu^91^ and Arg^298^ are the observations that these two residues are interchangeable without significant effect on the activity of NBCe1 (Chang et al., 2008), whereas single mutation on Glu^91^ or Arg^298^ markedly reduces the activity of NBCe1-A (Igarashi et al., 1999; Chang et al., 2008).

As discussed above, the Nt domain is critical for the transport activity the NCBTs. However, more structural and functional studies are required to demonstrate whether the Nt domain indeed contains a ${\text{HCO}}_{3}^{-}$ entry tunnel as hypothesized by Chang et al. More recently, Shnitsar et al. disagreed with the ${\text{HCO}}_{3}^{-}$ entry tunnel hypothesis based upon a study with human AE1 (Shnitsar et al., 2013). The authors proposed that the Nt domain of AE1, and likely other SLC4 family members, does not contain a substrate entry tunnel that is essential for the transport activity of the proteins. Their arguments include: (1) AE1 mutant carring R^293^S substitution—equivalent to the natural mutation R^298^S in NBCe1-A (Igarashi et al., 1999)—retains full transport activity (Shnitsar et al., 2013); (2) As aforementioned, removal of the entire cytoplasmic domain by either proteolytic treatment or recombinant DNA manipulation has no effect on the transport activities of the anion exchangers (Grinstein et al., 1978; Stewart et al., 2001; Shnitsar et al., 2013).

## Role of TMD in SLC4 transporters

The TMD of the SLC4 transporters is the structural component responsible for ion translocation across the plasma membrane. The fine 3D structure of the TMD of SLC4 transporters remains unknown. Several cryo-EM studies have been performed to investigate the 3D structure of AE1 (Wang et al., 1994; Yamaguchi et al., 2010a,b; Jiang et al., 2013). However, none of these cryo-EM structures has reached resolutions at atomic level. Due to the lack of 3D structure information, not too much is known about the molecular mechanisms underlying the ion translocation by the SLC4 transporters.

Consistent with the dimerization of the Nt domain observed in crystal structures (see discussion above in “Role of Nt domain in SLC4 transporters”), it is generally believed that the SLC4 transporters primarily exist as dimers in the plasma membrane (Kao et al., 2008; Yamaguchi et al., 2010b; Jiang et al., 2013). Of course, higher degree of oligomerization (e.g., tetramer) might also exist for the SLC4 transporters *in situ* in the cells. Functional study with NBCe1 shows that each monomer contains an ion translocation pathway independent of the other associated monomer (Kao et al., 2008). Based upon the putative topological models, mutational studies on AE1 and NBCe1 have identified multiple putative TMs that are likely involved in the formation of the ion translocation pathway across the plasma membrane. Such TMs include TM 1, 3, 5, 8, 13, and 14 (see Figure 2). As discussed below, these TMs contain specific residues likely lining along the cavity space of the ion translocation pathway. When replaced with cysteine, the positions of these residues are accessible by and sensitive to inhibition of sulfhydryl reagents, such as BM, MTS-TAMRA, (2-aminoethyl) methanethiosulfonate (MTSEA), [2-(trimethylammonium) ethyl] methanethiosulfonate (MTSET), and *para*-chloromercuribenzene sulfonate (pCMBS).

In the sections below, we will discuss the role of the TMD structural elements in the ion translocation by SLC4 transporters.

### TM1

TM1 is likely involved in the formation of ion translocation pore. Ala^428^, Ala^435^, Thr^442^ in TM1 of NBCe1-A are likely located on the same side of the putative helical wheel of TM1, contributing to the formation of the surface of the ion pathway (Zhu et al., 2009). NBCe1-A mutant with Ala^435^ or Thr^442^ replaced with cysteine is highly sensitive to inhibition by sulfhydryl reagents, such as MTSES, MTSET, and MTSEA. Thr^442^ is predicted to be located at the extracellular end of TM1 of NBCe1-A. It is intriguing that cysteine substitution of Thr^422^ in AE1 (homologous to Thr^442^ in NBCe1-A) has no effect on the activity of AE1 (Zhu et al., 2009).

Another line of evidence supporting the importance of TM1 in ion transport is that substitution of Ser^427^ with Leu in TM1 greatly reduces the intrinsic activity of NBCe1-A in *Xenopus* oocytes (Dinour et al., 2004; Li et al., 2005). S^427^L mutation in NBCe1 causes severe proximal renal tubular acidosis (pRTA) in human (Dinour et al., 2004). It is hypothesized that the substitution of Ser^427^ with Leu likely disturbs the orientation of TM1 (Zhu et al., 2013).

### TM3

TM3 is likely involved in the formation of ion translocation pore. Human AE1 mutants with Leu^468^ (corresponding to Val^488^ in NBCe1-A) or Phe^471^ (corresponding to Phe^491^ in NBCe1-A) in TM3 substituted by cysteine are highly sensitive to inhibition by pCMBS, suggesting that these two residues are lining along the ion pathway of AE1 (Barneaud-Rocca et al., 2013).

In NBCe1, two mutations of T^485^S and G^486^R in the putative TM3 are associated with severe pRTA in human (Horita et al., 2005; Suzuki et al., 2008). Although no effect on the membrane trafficking of NBCe1 in MDCK cells (Suzuki et al., 2008), both mutations cause a decrease by more then 50% in the transport activity of NBCe1-A when heterologously expressed in HEK293 cells (Suzuki et al., 2008, 2010). The mutations T^485^S and G^486^R likely cause great structural disturbation in the ion translocation pathway of NBCe1.

### TM4

The role of the putative TM4 in ion translocation is largely unknown. A missense mutation L^522^P in TM4 of NBCe1 causes cellular retention (Demirci et al., 2006; Suzuki et al., 2008, 2010). Therefore, it is practically difficult to examine the effect of this mutation on the transport function. Another missense mutation R^510^H at the extracellular end of TM4 causes a decrease by ~40% in the transport activity of NBCe1-A in human endothelial cell EVC304 (Igarashi et al., 1999; Suzuki et al., 2010).

### TM5

TM5 is likely involved in the formation of the ion translocation pore. In human AE1, cysteine substitution at Ile^528^, Phe^532^, or Glu^535^ in the putative TM5 substantially reduces the intrinsic transport activity. Mutants L^530^C and I^533^C are sensitive to inhibition by pCMBS, suggesting that these two residues are lining along the ion translocation pore (Barneaud-Rocca et al., 2013). In NBCe1-A, Asp^555^ (homologous to Glu^535^ in human AE1) in TM5 likely plays a role in determining the ion selectivity of the transporter (Yang et al., 2009).

Another line of evidence supportings that the putative TM5 contributes to the formation of ion translocation pore is that the extracellular ends of TM5 of some SLC4 members contain a binding motif (e.g., “KLIK” in AE1 and “KKMIK” in NBCe1) for stilbene disulfonate derivatives, such as 4,4′-diisothiocyanodihydrostilbene-2,2′-disulfonic acid (H_2_DIDS), 4-acetamido-4′-isothiocyanostilbene-2,2′-disulfonic acid (SITS), and 4,4′-diisothiocyanostilbene-2,2′-disulfonic acid (DIDS) (Kietz et al., 1991; Okubo et al., 1994; Lu and Boron, 2007). The stilbene reagents exhibit two phases of interaction with the transporters, one fast reversible involving ionic interaction with the Lys residues, the other slow irreversible involving covalent binding with the amino group of the Lys residues (Cabantchik and Rothstein, 1972; Bartel et al., 1989; Lu and Boron, 2007).

The presence of DIDS binding motif indicates that the extracellular end portion of TM5 is likely involved in the formation of the extracellular vestibule. It is proposed that the positive charges on the sidechains of the Lys residues are involved in the interaction with substrate anions during ion translocation by AE1 (Cabantchik and Rothstein, 1972). Consistent with this hypothesis, the anion permeability (assayed by ${\text{SO}}_{4}^{2-}$ flux) of AE1 in human erythrocytes is reduced by blocking the positive charges on the Lys residues either by binding of stilbene reagents or by increasing the solution pH to convert the siechain −${\text{NH}}_{3}^{+}$ to −NH_2_ (Cabantchik and Rothstein, 1972). However, inconsistent with this hypothesis, another study showed that replacing the two Lys residues in the DIDS binding motif with the neutral Asn has no significant effect on the anion exchange activity of mouse AE1 expressed in *Xenopus* oocytes, except that the mutant AE1 loses the capacity of irreversible inhibition by H_2_DIDS (Bartel et al., 1989). Additional observations seemingly inconsistent with the above hypothesis are that extracellular alkalinization, which would reduce the positive charges on the Lys residues, stimulates the anion exchange activities (assayed by Cl^−^ flux) of AE1-3 expressed in *Xenopus* oocytes (Zhang et al., 1996; Stewart et al., 2001, 2002, 2007c).

Interestingly, in human AE1, Lys^539^ at the outside end of TM5 and Lys^851^ at the outside end of TM13 react with the same H_2_DIDS molecule, suggesting that these two sites are in close proximity (Okubo et al., 1994).

### EL4

There are several line of evidences supporting the extracellular localization of the putative fourth extracellular loop EL4. Firstly, EL4 of AE1 contains an N-glycosylation motif “NSSA.” Note that, AE1 is the only SLC4 member that contains N-glycosylation motif in EL4. The N-glycosylation motifs are located on EL3 in all other SLC4 members. Secondly, introducing an N-glycosylation motif into the EL4 of a human NBCe1-A variant with all potential N-glycosylation motifs on EL3 removed results in slight N-glycosylation of the transporter (Chen et al., 2011). Thirdly, when replaced with cysteine residues, the positions following the N-glycosylation site in the putative EL4 of human AE1 are highly accessible to cysteine-reactive membrane impermeant reagent LYIA (Tang et al., 1998).

EL4 plays a critical role in determining the electrogenicity vs. electroneutrality of NCBTs (Chen et al., 2011). Replacing EL4 of NBCe1 with the counterpart of the electroneutral NBCn1 converts the electrogenic NBCe1 into electroneutral. The opposite manipulation converts an artificial electroneutral NCBT into electrogenic. It is proposed that EL4 is involved in the formation of the extracellular vestibule and likely contains binding sites for substrate ions (Chen et al., 2011).

Consistent with the functional significance of EL4 in NCBTs, the putative EL4 of AE2 contains determinants for the pH sensitivity of AE2 (Stewart et al., 2007a).

### TM8

TM8 is likely involved in the formation of ion translocation pore as revealed by studies with AE1 and NBCe1 (Tang et al., 1999; McAlear and Bevensee, 2006; Barneaud-Rocca et al., 2011). Surprisingly, TM8 appears to provide a hydrophobic surface lining along the ion translocation pore (Tang et al., 1999; McAlear and Bevensee, 2006). In human AE1, the hydrophobic surface appears to consist of Ala^666^, Ser^667^, Leu^669^, Leu^673^, Leu^677^, Leu^680^ (Tang et al., 1999). When replaced with cysteine, positions 673 and 677 of human AE1 are highly accessible to sulfhydryl reagents pCMBS and MTSEA (Tang et al., 1999). Consistently, NBCe1 mutant L^750^C (corresponding to L^677^C in AE1) is highly sensitive to inhibition by pCMBS and MTSEA (McAlear and Bevensee, 2006).

Two additional positions (Ile^684^ and Ile^688^ in human AE1) in IL4 immediately following the intracellular end of TM8 are also important for ion transport. AE1 mutants with Ile^684^ or Ile^688^ replaced by Cys are sensitive to inhibition by sulfhydryl reagents. In some models, it is proposed that the region beyond Ile^688^ (corresponding to Ile^761^ in NBCe1-A) forms an extended portion of the helix of the putative TM8 (Tang et al., 1999; Barneaud-Rocca et al., 2013; Bonar et al., 2013; Figure 2B).

A specific position in TM8, Glu^681^ in AE1 (corresponding to Asp^754^ in NBCe1-A), is of particular interest. This Glu residue is conserved in all three established AEs but is replaced by Asp in all five NCBTs plus AE4. This Glu residue plays a role in determining the substrate selectivity of AE1 and AE2 as well as the pH sensitivity of AE2 (Sekler et al., 1995). In NBCe1-A, cysteine substitution of Asp^754^ inactivates the transporter (McAlear and Bevensee, 2006). Interestingly, replacing with Asp of this Glu in AE1 and AE2 substantially decreases the anion exchange activity (Sekler et al., 1995).

The significance of the Glu residue in ion translocation is also evidenced by the effect of chemical modification by the so-called Woodward's reagent K on AE1 (Jennings and Anderson, 1987; Jennings and Al Rhaiyel, 1988; Jennings and Smith, 1992). Treatment with the Woodward's reagent K followed by reduction with NaBH_4_ converts the sidechain carboxyl group of Glu residue to alcohol. This conversion inhibits the monovalent anion exchange by AE1 in human red blood cells (Jennings and Al Rhaiyel, 1988).

### TM13-TM14

The sequence assignments of the last two TMs (TM3 and TM14) are basically consistent between model A and model B, except for some minor differences in the sequence boundary (Figure 2). These two TMs are connected by a very short extracellular loop. Here, we discuss these two TMs together.

The significance of these two TMs in the structure and function of the transporters is supported by cysteine scanning mutagenesis study. In AE1, the region TM13-TM14 contains multiple sites that, when replaced with cysteine residues, are sensitive to inhibition by sulfydryl reagents pCMBS, MTSEA, and MTSET to varying extent. Such sites include positions Val^849^ and Val^850^ (corresponding to Leu^923^ in NBCe1-A) at the extracellular end of the putative TM13, Ser^852^ and Thr^853^ in the putative EL7, and Leu^857^ (corresponding to Ile^930^ in NBCe1-A), Ala^858^, Phe^861^, Val^862^, and Ile^863^ in the putative TM14. The observations suggest that these redidues in TM13 and TM14 is important for ion translocation. The residues accessible to sulfhydral reagents are likely involved in the formation of the ion translocation pore. The inactivation of Cl^−^/${\text{HCO}}_{3}^{-}$ exchange activity by cysteine substitutions at any of Met^833^, His^834^, Phe^836^, Thr^837^, Gln^840^ in the putative TM13, and Lys^851^ in the putative EL7 also support the functional and/or structural significance of TM13 in AE1 (Zhu and Casey, 2004).

The extracellular ends of TM13 and TM14 plus the extracellular loop in between presumably contribute to the formation of the extracellular vestibule of the SLC4 transporters. Consistent with this hypothesis is that, Lys^539^ (at the extracellular end of TM5) and Lys^851^ in human AE1 (corresponding to Lys^559^ in TM5 and Lys^924^ in TM13 of NBCe1-A) react with the same DIDS molecule (Okubo et al., 1994), indicating that the extracellular ends of TM5 and TM13 are juxtaposed in the 3D structure of the transporter.

## Role of Ct domain in SLC4 transporters

Compared to the large Nt domain and the TMD, the Ct domain of the SLC4 ${\text{HCO}}_{3}^{-}$ transporters is small, accounting for only 3-10% of the entire polypeptide, depending on specific variants of specific SLC4 members. The anion exchangers have a fairly short Ct tail with ~40 residues only, whereas the longest Ct of NBCn1 variants with cassette ε (*aka* cassette III) contains ~130 residues. The initial portion of the Ct domain (Ct-CR1) right following the TMD is highly conserved among all SLC4 family members, whereas the last portion (Ct-VR1) of the Ct domain is highly variable and has little similarity among different members.

The Ct domain is important for the function of SLC4 transporters in the following three perspectives. Firstly, the Ct domain is indispensable for the functional expression, likely the membrane trafficking, of the SLC4 transporters. Removal of partial or the entire Ct domain greatly reduces the surface expression of NBCe1 in *Xenopus* oocytes, suggesting that the Ct domain is important for the membrane trafficking of NBCe1 (McAlear et al., 2006). Moreover, removal of the Ct portion abolishes the Cl^−^/${\text{HCO}}_{3}^{-}$ exchange activity of AE1 expressed in *Xenopus* oocytes (Dahl et al., 2003).

Secondly, as discussed in section “Structural variations in SLC4,” the Ct domain of some SLC4 transporters (e.g., NBCn1 and NDCBE) contains OSEs that could affect the intrinsic activity of the transporters.

Thirdly, the Ct domains of some SLC4 family transporters contain binding sites for protein partners. The Ct-CR1 of some members contain a conserved motif (typically “LD[A/D][D/L][D/M/E]” enriched with acidic residues) which is likely the binding site of carbonic anhydrase. Studies have provided evidences showing that the Ct of AE1 interacts with carbonic anhydrase CAII (Vince and Reithmeier, 1998, 2000). However, the interaction with CAII was questioned by Piermarini et al who showed evidence that the binding of the Ct of AE1, NBCe1, and NDCBE to CAII appears to be an artifact of GST (Piermarini et al., 2007). Finally, the Ct ends of specific variants of some SLC4 members contain typical binding motif of PDZ-domain containing scaffold proteins (Cowan et al., 2000; Park et al., 2002; Pushkin et al., 2003; Lee et al., 2006, 2012a, 2014; for review, see Boron et al., 2009; Parker and Boron, 2013). At least some of these interactions have been shown to affect the function of the SLC4 transporters. Interaction with PSD-95 stimulates the cation conductance, but has no effect on Na^+^/${\text{HCO}}_{3}^{-}$ cotransport activity of NBCn1 expressed in *Xenopus* oocytes (Lee et al., 2012a). Interaction with syntrophin γ2 stimulates both the conductance and Na^+^/${\text{HCO}}_{3}^{-}$ cotransport activity of NBCn1 in *Xenopus* oocytes (Lee et al., 2014). When expressed in mouse fibroblast 3T3 cells, the activity of NBCn2-C is down-regulated by protein kinase A via a PDZ-protein dependent manner (Lee et al., 2006).

## Structural variations in SLC4 transporters

All genes encoding the SLC4 family ${\text{HCO}}_{3}^{-}$ transporters are able to express multiple variants, producing a great extent of structural diversity in the products of these genes. Molecular clonings during the past decades have identified a great number of variants of these SLC4 members. As summarized in Figure 5, the structural variations in SLC4 family products include alternative Nts (distinct extreme Nt ends), optional cassettes, and alternative Cts (distinct extreme Ct ends).

The alternative Nts arise from two different types of sources: (1) transcription using distinct promoters, e.g., the generation of alternative Nts of NBCe1-A vs. NBCe1-B (Abuladze et al., 2000); (2) alternative splicing of cassette exons in the 5′ portion of the messenger RNAs, e.g., the generation of alternative Nts of NBCn1-Q vs. NBCn1-R (Wang et al., 2015). Generally, the alternative Nts have little similarity among different SLC4 family members and different variants of the same member.

The optional cassettes are derived from alternative splicing of cassette exons. As shown in Figure 5, most of the known optional cassettes are localized in the Nt and Ct domains. The only exception is cassette ϵ of NBCe2 which is localized in the TMD. So far, optional cassettes have been identified in NCBTs and AE4 only.

The alternative Cts arise from alternative splicing of cassette exons in the 3′ region of the messenger RNAs. Alternative splicing of such cassette exons causes a frame shift, resulting in the expression of distinct Ct ends. Note that, alternative Cts are just identified in the variants of NCBTs, but not in those of the anion exchangers.

The alternative Nts, the optional cassettes, and the alternative Cts are collectively referred to as optional structural elements (OSEs) in the present article. These OSEs play critical roles in the functional modulation of the transporters. Functionally, the OSEs could contain structural determinants establishing the biophysical properties, e.g., the intrinsic activity and pH sensitivity, of the transporters. Moreover, the OSEs could regulate the transport function by interacting with protein partners.

Firstly, the intrinsic transport activity (or single molecular activity) of different variants of some SLC4 members can vary greatly, e.g., NBCe1-A vs. NBCe1-B/C (McAlear et al., 2006). Intrinsic transport activity is an index of single molecular transport rate (or turnover number), a fundamental biophysical property of a transporter. Some variable regions in the Nt and Ct domains exhibit stimulatory or inhibitory effect on the intrinsic activity of the transporters. However, the mechanism underlying the stimulatory or inihitory effect of the OSEs on the transport activities largely remains to be addressed.

Secondly, the presence or absence of OSEs could affect the pH sensitivity of the transporters, another important biophysical property of the acid-base transporters. Anion exchangers AE2 and AE3 are highly sensitive to changes in intracellular as well as extracellular pH, whereas AE1 is much less sensitive (Zhang et al., 1996; Stewart et al., 2007c; for review, see Stewart et al., 2007b). When heterologously expressed in *Xenopus* oocytes, cultured Chinese hamster ovary cells, or human embryonic kidney cells HEK293, both AE2 and AE3 are strongly activated by alkalosis and inhibited by acidosis (Lee et al., 1991; Jiang et al., 1994; Zhang et al., 1996; Stewart et al., 2001, 2002). As will be discussed below, the OSEs of AE2 contains structural elements affecting the pH sensitivity of these transporters.

Thirdly, the OSEs in the Nt and Ct domains of SLC4 transporters often contain binding sites for interacting partners. The Nt-VR1 of eAE1 contains structural determinants for the binding of cytoskeletal proteins. Nt-VR1 of NBCe1-B/C, NBCn1, and NBCn2 contains binding site for IRBIT, which is an inositol trisphosphate (IP3)-receptor (IP3R) binding protein released with IP3 (Ando et al., 2003). Cassette β (*aka* cassette II) of NBCn1 contains binding site for calcineurin Aβ (CnAβ). Finally, as just discussed in the previous section, the Ct ends of variants of several SLC4 members contain binding motif for PDZ-domain containing scalfold proteins. Some of these protein interactions have great effect on the activities of the transporters.

In the following sections, we will discuss the structural variations and their potential consequences on the functions of each of the SLC4 family ${\text{HCO}}_{3}^{-}$ transporters.

### AE1 (SLC4A1)

AE1 (*aka* Band 3), was the first to be cloned in the SLC4 family, originally from murine spleen (Kopito and Lodish, 1985), and then from human erythrocytes (Lux et al., 1989). *SLC4A1* encoding AE1 has two alternative promoters (Sahr et al., 1994; Schofield et al., 1994), regulating the expression of two variants with alternative Nts: the erythrocyte variant eAE1 (Kopito and Lodish, 1985; Lux et al., 1989) and the kidney variant kAE1 (Kollert-Jöns et al., 1993). The initial portion (Nt-VR1, 65 aa in human) of eAE1 is absent in kAE1. AE1 variants have no structural variation in the TMD and Ct domain.

Nt-VR1 appears to be intrinsically disordered and is largely invisible in the crystal structures of the Nt domain of AE1 (Zhang et al., 2000; Shnitsar et al., 2013). Nt-VR1 is dispensable for the anion-exchange activity of AE1, as kAE1 lacking the whole Nt-VR1 is active in mediating Cl^−^/${\text{HCO}}_{3}^{-}$ exchange when heterologously expressed in *Xenopus* oocytes (Bruce et al., 1997; Fry et al., 2012). Actually, as discussed in section “Role of Nt domain in SLC4 transporters,” the whole Nt domain is not necessary for the transport activity of AE1. The role of Nt-VR1 in the structure and function of AE1 remains to be addressed.

### AE2 (SLC4A2)

In mouse, five AE2 variants (AE2a, AE2b1, AE2b2, AE2c1, and AE2c2) have been identified, whereas in human, only the first three have been identified. The five mouse AE2 variants are derived from three different promoters, designated as a, b, and c (Wang et al., 1996; Lecanda et al., 2000; Medina et al., 2000). As shown in Figure 5, variants AE2a (first 17 aa), AE2b1 (first 3 aa), and AE2b2 (first 8 aa) are only slightly different from each other in the initial Nt ends, whereas AE2c1 is truncated in the Nt by 198 aa compared to AE2a. AE2c2 contains an additional 32 residues at the Nt end compared to AE2c1. Except for the initial Nt ends, AE2 variants have no additional structural variations in other regions.

The activities of AE2 variants are highly sensitive to acute changes in intracellular as well as extracellular pH. For example, mouse AE2a, AE2b1, AE2b2, and AE2c1 are all steeply sensitive to regulation by pH when heterologously expressed in *Xenopus* oocytes (Kurschat et al., 2006). Compared to the other AE2 variants, the activation of AE2c1 requires more alkaline pH. These observations suggest that the optional region omitted in the Nt of AE2c1 contains determinants for the difference in pH sensitivities of different AE2 variants. Further studies have shown that, removal of the first 99 residues of Nt-VR1 of mouse AE2a has little effect on the pH sensitivity of AE2-mediated anion-exchange activity (Zhang et al., 1996; Stewart et al., 2001; Kurschat et al., 2006). The key structural element responsible for the differences in the pH sensitivities of AE2a and AE2c1 lies in the Glu-rich sequence 120-150 (Kurschat et al., 2006).

Note that, although not discussed here, the non-variable region of Nt domain and the TMD of AE2 also contains structural determinants for the pH sensitivity (Stewart et al., 2002, 2007a,c; for review, see Alper, 2009).

### AE3 (SLC4A3)

*SLC4A3* encoding AE3 has two alternative promoters, responsible for the expression of two full-length variants: bAE3 and cAE3. bAE3, literally meaning “the brain variant,” is expressed under the control of the distal promoter, whereas cAE3 (literially “the cardiac variant”) is expressed under the control of the proximal promoter (Yannoukakos et al., 1994). Note that the non-menclatures of bAE3 and cAE3 variants are only historically meaningful as they are also expressed in other tissues, e.g., bAE3 in the intestine (Alrefai et al., 2001), bAE3 and cAE3 in the kidney (Kampik et al., 2014). The first 271 aa of bAE3 is unique compared to the first 73 aa of cAE3. Except for the initial Nt ends, the AE3 variants have no additional structural variations in other regions.

As shown in Figure 5, the first 270 aa of the Nt of bAE3 is unique compared to the first 73 aa of cAE3. Note that, the Nt-VR1 (the red plus yellow regions) of bAE3 shares high sequence homology with that of AE2a. Moreover, the boundary of exons encoding Nt-VR1 of bAE3 is identical to that of AE2a. These facts suggest that the Nt-VR1 of AE2a and bAE3 have been elvoved from a common ancestor. Except for the difference in the extreme Nt ends, no OSE has been identified in other regions of AE3. The functional consequence of structural variation in AE3 remains unclear.

### AE4 (SLC4A9)

Compared to the other eight members of SLC4 family ${\text{HCO}}_{3}^{-}$ transporters, AE4 is less well characterized. Several AE4 variants have been reported in GenBank, shown in Figure 5 are only two variants: AE4a and AE4b originally identified from rabbit (Tsuganezawa et al., 2001). Other variants are not included here as they are not well characterized and even questionable, e.g., containing large deletion in the TMD. Due to alternative usage of a cryptic splicing acceptor site at the 5′ portion of the coding exon, AE4b lacks a 16-aa cassette (cassette ε) in Nt-VR3 compared to AE4a. Although not identified, DNA sequence alignment shows that the potential cryptic splicing site for this cassette is conserved in all five genes encoding the NCBTs.

### NBCe1 (SLC4A4)

NBCe1 was originally identified from the renal proximal tubule of salamander (Boron and Boulpaep, 1983). It was the first to be cloned among the five NCBTs (Romero et al., 1997). *SLC4A4* encoding NBCe1 has two alternative promoters and two cassette exons. *SLC4A4* is able to express at least five different variants, namely, NBCe1-A through -E. NBCe1-A/D are expressed under the control of the proximal promoter, whereas NBCe1-B/C/E are expressed under the control of the distal promoter.

The five NBCe1 variants contain three structural variations: the alternative Nts, optional cassette α (*aka* cassette I) in the Nt domain, and the alternative Cts. As shown in Figure 5, the initial 41 aa of Nt-VR1 of NBCe1-A/D is distinct from the initial 85 aa of NBCe1-B/C/E due to alternative transcription using different promoters of *SLC4A4* (Abuladze et al., 2000). The 9 aa optional cassette α in Nt-VR2 arises from alternative splicing of the 3′ portion of exon 6 of *SLC4A4* (Liu et al., 2011). The last 61 aa of the Ct domain of NBCe1-C is unique compared to the last 46 aa of the Ct domain of NBCe1-A/B/D/E. The differences in the alternative Cts of NBCe1 are due to alternative splicing of exon 24 of *SLC4A4* (Bevensee et al., 2000). This exon 24 contains a stop codon. Inclusion of exon 24 in the messenger RNAs results in the production of the short Ct of NBCe1-A/B/D/E. Exclusion of exon 24 in the messenger RNA results in the expression of the long Ct of NBCe1-C.

The differences in the initial portion of Nt-VR1 are particularly important for the functional modulation of NBCe1. Firstly, this region plays a critical role in establishing the intrinsic activity of NBCe1 variants. Specifically, the unique region in Nt-VR1 of NBCe1-A is stimulatory, whereas the unique region in Nt-VR1 of NBCe1-B/C is inhibitory to the intrinsic activity of the transporters (McAlear et al., 2006). The stimulatory or inhibitory effect of Nt-VR1 is so profound that the intrinsic activity of NBCe1-A is about 4-5 times higher than those of NBCe1-B/C when heterologously expressed in *Xenopus* oocytes (McAlear et al., 2006). However, it remains mystic about the molecular mechanisms underlying the stimulatory vs. inhibitory effects of unique regions of Nt-VR1 of NBCe1 variants.

Secondly, the unique region of Nt-VR1 of NBCe1-B/C contains structural determinants for the binding of IRBIT (Shirakabe et al., 2006). IRBIT interacts with inositol trisphosphate (IP3)-receptor (IP3R) but is released from the latter upon the binding of IP3 to IP3R (Ando et al., 2003). IRBIT can substantially stimulate the transport activity of NBCe1-B/C to about the same level of the activity of NBCe1-A (Shirakabe et al., 2006; Thornell et al., 2010; Lee et al., 2012b). In NBCe1-B, the structural determinant essential for the binding of IRBIT is located in the first 19 aa of Nt-VR1 and is distinct from the structural determinant for the auto-inhibitory effect of Nt-VR1 (Lee et al., 2012b).

The functional consequence of cassette I in Nt-VR2 remains to be investigated. The difference in the Ct ends of NBCe1 variants has no significant effect on the transport activity when expressed in *Xenopus* oocytes (McAlear et al., 2006). Finally, the long Ct of NBCe1-C contains a typical PDZ-binding motif.

### NBCe2 (SLC4A5)

NBCe2 was originally cloned from human testes (Pushkin et al., 2000). It is the second established electrogenic Na^+^/${\text{HCO}}_{3}^{-}$ cotransporter of the SLC4 family. *SLC4A5* encoding NBCe2 contains two alternative promoters (Stütz et al., 2009; Fukuda et al., 2013). So far, seven NBCe2 variants have been reported in GenBank, designated as NBC4a-g (referred to as NBCe2-a through -g here). Shown in Figure 5 are only NBCe2-a, -c, and -g. NBCe2-c and NBCe2-g have been functionally characterized (Sassani et al., 2002; Virkki et al., 2002; Fukuda et al., 2013). Although not tested, NBCe2-a likely represent an active transporter. As discussed elsewhere (Parker and Boron, 2013; Romero et al., 2013), the other four NBCe2 variants are questionable as they contain unusual deletions in the TMD.

The three NBCe2 variants shown in Figure 5 contain two OSEs: (1) Nt-VR1 that is present in NBCe2-a/c and truncated in NBCe2-g; (2) cassette ε of 16 aa in TMD that is present in NBCe2-a and absent in NBCe2-c and -g. Cassete ε is derived from alternatives splicing of cassette exon 27 (see review by Parker and Boron, 2013). Reminiscent of kAE1, NBCe2-g lacking the entire Nt-VR1 is well expressed on the plama membrane and functionally active (Fukuda et al., 2013; Wang et al., 2015), demonstrating that Nt-VR1 is dispensable for the transport activity of NBCe2. The functional significance of cassette ε remains unclear.

### NBCn1 (SLC4A7)

NBCn1 was the first characterized electroneutral Na^+^/${\text{HCO}}_{3}^{-}$ cotransporter (Pushkin et al., 1999; Choi et al., 2000). *SLC4A7* encoding NBCn1 has two alternative promoters and as many as six major cassette exons (Liu et al., 2013a; Wang et al., 2015). So far, 18 full-length NBCn1 variants have been identified, designated as NBCn1-A through -R. In addition, *SLC4A7* is able to express two types of special variants containing just the Nt domain: NBCn1-e/g and NBCn1-c/h (Liu et al., 2013a). Herein, the term “full-length” is used to refer to the variants of SLC4 members that contain all three major domains of Nt, TMD, and Ct, differing from the product like NBCn1-e/g containing just the isolated Nt domain.

These NBCn1 variants have four different types of initial Nt ends, commonly referred to as “MEAD,” “MERF,” “MIPL,” or “MDEL,” each representing the first four residues of the corresponding Nt (Pushkin et al., 1999; Choi et al., 2000; Liu et al., 2013a; Wang et al., 2015). MEAD-NBCn1 (including full-length NBCn1-B/C/D/E/G/H/I/O/P and NBCn1-e/g and -c/h containing just the isolated Nt domain) is expressed under the control of the distal promoter, whereas MERF-NBCn1 (including NBCn1-A/F/J/K/L/M/N), MIPL-NBCn1 (NBCn1-R), and MDEL-NBCn1 (NBCn1-Q) are expressed under the control of the proximal promoter of *SLC4A7* (Liu et al., 2013a; Wang et al., 2015). As shown in Figure 5, the initial Nt of MEAD-, MERF-, and MIPL-NBCn1 are just slightly different from each other, whereas MDEL-NBCn1 is truncated by Nt-VR1 compared to the other three types of NBCn1 variants.

In addition to the differences in the initial Nt ends, NBCn1 contains four optional cassettes: cassettes α, β, and γ (*aka* cassettes I, II, and IV) in the Nt domain and cassette ζ (*aka* cassette III) in the Ct domain. Cassette β of NBCn1 is the largest (123 aa in rodent and 124 aa in human) among all reported optional cassettes in the SLC4 family ${\text{HCO}}_{3}^{-}$ transporters. Cassette α is present in NBCn1-A/B/D/E/F/G/J/N/P/Q and NBCn1-e/g. Cassette β is present in NBCn1-A/B/C/D/K/H/R and NBCn1-c/h. Cassette γ is present in NBCn1-M/N/O/P. Cassette ζ is present in NBCn1-C/D/G/I/J/L/M/N/O/P/Q.

Except for NBCn1-e/g and -c/h with just the Nt domain, all full-legnth NBCn1 variants have the same extreme Ct ends.

The structural differences in the initial Nts of MEAD-, MERF-, and MIPL-NBCn1 appear to have no substantial effect on the intrinsic activity of NBCn1 (Liu et al., 2013a; Wang et al., 2015). NBCn1-Q lacking the entire Nt-VR1 has only minimal transport activity minimal in *Xenopus* oocytes although it can be sufficiently expressed on the plasma membrane, suggesting that Nt-VR1 is important for the intrinsic activity of NBCn1 (Wang et al., 2015). As indicated in Figure 5, the Nt-VR1 of NBCn1 is modestly homologous to the Nt-VR1 of NBCe1-B/C. Similar to NBCe1-B/C, the Nt-VR1 of NBCn1 likely contains binding determinants for IRBIT, as evidenced by a preliminary observation that coexpressing IRBIT increases the functional expression of rat NBCn1 in *Xenopus* oocytes (Parker et al., 2007b).

Among the optional cassettes, cassette α appears to have no significant effect on the intrinsic activity of NBCn1, whereas cassettes β, γ, and ζ have stimulatory effects on the intrinsic activity of NBCn1 heterologously expressed in *Xenopus* oocytes (Liu et al., 2013a). Cassette β is of particular interest for the functional regulation of NBCn1 in asmuch as it contains binding determinants for calcineurin CnAβ (Parker and Boron, 2008), a Ca^2+^-calmodulin activated Ser/Thr phosphatase (Rusnak and Mertz, 2000). The structural element essential for the binding of CnAβ lies in the last 57 residues of cassette β of NBCn1 (Parker and Boron, 2008). The binding of CnAβ stimulates the activity of NBCn1 in the vascular smooth muscle cells (Danielsen et al., 2013), where NBCn1 plays a critical role in the regulation of intracellular pH (Boedtkjer et al., 2011).

### NBCn2 (SLC4A10)

*SLC4A10* encoding NBCn2 contains three alternative promoters (at least in rat *Slc4a10*) and as many as seven cassette exons (Liu et al., 2013b; Wang et al., 2015). So far, *SLC4A10* is known to express 15 “full-length” NBCn2 variants (NBCn2-A through -N, plus rb3NCBE) as well as a specific variant rb7NCBE containing just the Nt domain. These variants have three different initial Nt ends starting with MEIK- (NBCn2-A through -D plus rb3NCBE and rb7NCBE), MCDL- (NBCn2-E through -J), and MHAN-NBCn2 (NBCn2-K through -N), respectively. MEIK-NBCn2 is expressed under the control of the most distal promoter P1. MCDL-NBCn2 could be expressed from either the middle promoter P2 or the proximal promoter P3. Finally, MHAN-NBCn2 is expressed under the control of the middle promoter P2 (Wang et al., 2015).

The initial Nt ends of MEIK- and MCDL-NBCn2 are just slightly different from each other. Note that, there is a species-specific variation in the Nt of the variants designated as “MCDL-NBCn2” here (Liu et al., 2013a). The initial Nt of the corresponding mouse variants start with “MQPG” rather than “MCDL.” Mouse MQPG-NBCn2 variants are truncated by 13 residues compared to the rat counterparts (Liu et al., 2013b). The structural variations in the initial Nts of MEIK- vs. MCDL- vs. MQPG-NBCn2 have no significant effect on the functional expression of NBCn2 expressed in *Xenopus* oocytes (Liu et al., 2013b). MHAN-NBCn2 is truncated by the entire Nt-VR1 plus about half of Nt-CR1 compared to other three full-length NBCn2 variants. This truncation causes severe cellular retention of NBCn2 when heterologously expressed in mammalian cell line neuro-2A (Wang et al., 2015).

The Nt domain of NBCn2 contains one major optional cassettes, i.e., cassette γ (*aka* cassette A in NBCn2). Cassette γ is present in NBCn2-B/D/F/H/J/L/N and “rb3NCBE.” The functional consequence of the presence vs. absence of cassette γ in NBCn2 remains unclear. In addition, the Nt domain of NBCn2 contains one minor variation, i.e., the optional inclusion of a single residue Ala^256^ (numbering according to MEIK-NBCn2) due to alternative usage of a cryptic splicing acceptor site. The optional inclusion of this single Ala residue appears to be random and presumably has no significant effect on the structure and function of NBCn2.

In the Ct domain, NBCn2 contains an optional cassette ζ (*aka* cassette C, 39 aa in length; see Wang et al., 2015). Cassette ζ is absent in NBCn2-M and -N. The functional consequences of the presence vs. absence of cassette ζ on the structure and function of NBCn2 remain to be investigated.

As shown in Figure 5, the known NBCn2 variants contain five different Ct ends: (1) NBCn2-C/D/G/H/K/L/M/N have the longest Ct with a typical PDZ binding motif “ECTL”; (2) NBCn2-A/B/E/F have a short Ct ending with “SPSS”; (3) NBCn2-I/J have a short Ct ending with “RS” (Liu et al., 2013b); (4) The Ct of rb3NCBE is truncated by the Ct-VR1; (5) rb7NCBE with just the Nt domain contains a unique Ct end. The differences in the first three types of Ct ends arise from the alternative splicing of the so-called cassette B (the exon right after the one encoding cassette ζ) of *SLC4A10* (Liu et al., 2013b), whereas the truncation in the Ct of rb3NCBE is due to the omission of cassettes B and C plus the exon procedding cassette ζ (see review by Parker and Boron, 2013). The functional relevance of the variations in the Ct ends of NBCn2 remains to be elucidated.

Finally, it is worth to note that, when co-expressed, the isolated Nt domain of NBCn2 is able to interact with MHAN-NBCn2 and promotes the membrane trafficking of the latter in heterologous expression system (Wang et al., 2015).

### NDCBE (SLC4A8)

*SLC4A8* encoding NDCBE contains two alternative promoters and as many as four cassette exons (Parker et al., 2008). So far, five full-length NDCBE variants, NDCBE-A through -E, have been identified. In addition, *SLC4A8* is able to produce a specific variant “THYMU3021755” with just the isolated Nt domain. NDCBE-A/B and “THYMU3021755” are expressed under the control of the proximal promoter, whereas NDCBE-C/D/E are expressed under the control of the distal promoter of *SLC4A8*. Except for the variations in the Nt and Ct ends, no optional cassette has been identified in the known NDCBE variants.

As shown in Figure 5, the known NDCBE variants have three different Nt ends: (1) NDCBE-A/B and THYMU3021755 containing the isolated Nt domain have a unique Nt end of 16 aa compared to NDCBE-E; (2) NDCBE-C/D are truncated at the Nt by 54 residues compared to NDCBE-A/B; (3) NDCBE-E has a unique Nt end of 43 aa compared to NDCBE-A. The structural variation in the Nt domain appears to have no significant effect on the functional expression of NDCBE in *Xenopus* oocytes (Parker et al., 2008).

The NDCBE variants have three different Ct ends: (1) The last 66 residues of the full-length NDCBE-A/C is unique compared to NDCBE-B/D/E; (2) The last 17 residues of NDCBE-B/D/E is unique compared to NDCBE-A/C; (3) The last 15 residues of THYMU3021755 with the isolated Nt domain is unique compared to the full-length variants. The differnce in the Ct ends of the full-length NDCBE arises from alternative splicing of exon 25 of *SLC4A8* (Parker et al., 2008). The unique 17 aa of the Ct end of NDCBE-B/D has inhibitory effect on their functional expression in *Xenopus* oocytes (Parker et al., 2008).

## Concluding remarks

Since the identification of the erythrocytic AE1 more than four decades ago, our knowledge about the SLC4 family ${\text{HCO}}_{3}^{-}$ transporters have been expanded greatly. The molecular cloning of a series of expression variants of the SLC4 genes has revealed many OSEs in the transporters. Some of these OSEs have been demonstrated to have important structural and functional relevances. Following the determination of the gross membrane organization of the SLC4 transporters, great advances have been made in understanding the topological structure of the TMD, although some major issues remain unsettled. Based upon the proposed topological models, mutational and functional studies have identified a number of structural elements important for the function of the trasnsporters. Particularly, some key transmembrane segments have been identified that are likely involved in the formation of ion translocation pathway. Moreover, as have been extensively reviewed elsewhere (Stewart et al., 2007b; Parker and Boron, 2013), genetics studies have identified a series of mutations in the SLC4 transporters, particularly in AE1 and NBCe1. The results from these studies shed important light for understanding the mechanism underlying the ion translocation by the ${\text{HCO}}_{3}^{-}$ transporters.

In future studies on the structure and function of the SLC4 transporters, the following issues are of particular interest to investigators. Firstly, although the crystal structure of the soluble Nt domain of some members have been reported, some major issues remain to be addressed regarding the role of the large Nt domain in the ion translocation. Why the Nt domains of AEs are not necessary, whereas those of NCBTs are indispensible for the ion transport activities? Except for their regulatory roles for the transporters, are the Nt domains also directly involved in the ion translocation process, e.g., being a part of the ion translocation pathway as suggested by Chang et al. (2008)?

Secondly, the fine 3D structure of the TMD of the SLC4 transporters remains mystic. Cryo-EM could be a promising strategy to resolve this issue, given the great progresses in the development of cryo-EM technology and its application in determining the near-atomic level structures of highly complex macromolecules including integral membrane proteins during the past years (Kühlbrandt, 2014). Red blood cell membrane could be an ideal system for cryo-EM study due to the extremely high abudance of AE1 in erythrocytic membrane.

Thirdly, as secondary active transporters, the molecular mechanism underlying the substrate coupling during ion transport by SLC4 proteins remains mystic. It remains unclear how the stoichiometry of the SLC4 transporters is determined. The stoichiometry of some electrogenic NCBTs, e.g., NBCe1-A, can be switched between 3:1 and 2:1 (${\text{HCO}}_{3}^{-}$ over Na^+^) depending on the expression systems or in response to specific stimulus (Gross et al., 2001). What is the mechanism underlying such conversion of the transport stoichiometry?

Fourthly, what is the structural basis for the differences in the Na^+^-dependence vs. Na^+^-independence of the ion transport by the SLC4 proteins? Are there fundamental differences in the structures of TMDs (e.g., the number and orientation of transmembrane helices in the topology structure) between the AEs and NCBTs? Is NDCBE (SLC4A8) a hybrid of anion exchanger and Na^+^/${\text{HCO}}_{3}^{-}$ cotransporter?

Answering these questions will require studies involving structural, biophysical, and functional approaches. It is no doubt that the knowledge about the structure and function of the SLC4 family transporters derived from these studies will greatly enhance our understanding about the physiology of these proteins. The knowledge about structure and function will also be of high relevance to clinical and pharmaceutical studies.

## Author contributions

YL and LC wrote the manuscript. JY contributed critical discussions for the preparation of the manuscript.

### Conflict of interest statement

The authors declare that the research was conducted in the absence of any commercial or financial relationships that could be construed as a potential conflict of interest.
