# Supplementary material for: Structure and Function of SLC4 Family HCO3- Transporters
Source: Front Physiol. 2015 Dec 1;6:355. doi: 10.3389/fphys.2015.00355 (PMC4664831; doi:10.3389/fphys.2015.00355)
Supplement: Supplementary file 1 [file Table1.PDF]

Supplemental Table S1. Sequence used for alignment in Figure 5.

Gene	Variant	Accession#	Species	Note
SLC4A1	eAE1	NP_000333.1	Human	erythrocyte
	kAE1	AAH85748.1	Rat	kidney
SLC4A2	AE2a	NP_003031.3	Human	
	AE2b1	NP_001186623	Human	
	AE2b2	NP_001186622	Human	
	AE2c1	AAG23157	Mouse	
	AE2c2	AAG23158	Mouse	
SLC4A3	bAE3	AAA50748.1	Human	brain
	cAE3	AAG25583	Mouse	cardiac
SLC4A4	NBCe1-A	NP_003750.1	Human	
	NBCe1-B	NP_001091954.1	Human	
	NBCe1-C	NP_001128214.1	Human	
	NBCe1-D	ADP37962.1	Mouse	
	NBCe1-E	ADN95183.1	Mouse	
SLC4A5	NBCe2-a	NP_067019.3	Human	
	NBCe2-c	NP_597812.1	Human	
	NBCe2-g	BAM73282.1	Rat	
SLC4A7	NBCn1-A	NP_003606.3	Human	
	NBCn1-B	AFR46591.1	Mouse	
	NBCn1-C	AFR46592.1	Mouse	
	NBCn1-D	AFR46593.1	Mouse	
	NBCn1-E	ACH61961.1	Human	
	NBCn1-F	NP_001245309	Human	
	NBCn1-G	NP_001245308.1	Human	
	NBCn1-H	ACH61958.1	Human	
	NBCn1-I	ADC92004.1	Mouse	
	NBCn1-J	ADO51787.1	Mouse	
	NBCn1-K	ADO51788.1	Mouse	
	NBCn1-L	AFB82586.1	Mouse	
	NBCn1-M	AFI43934.1	Mouse	
	NBCn1-N	AFB82538.1	Mouse	
	NBCn1-O	AFI43933.1	Mouse	
	NBCn1-P	AFI43932.1	Mouse	
	NBCn1-Q	AGX13876.1	Mouse	
	NBCn1-R	AGX13877.1	Mouse	
	NBCn1-e/g	ACI24741.1	Human	
		ACI24742.1	Human	
	NBCn1-c/h	ACI24740.1	Human	
		ADC32649.1	Human	
SLC4A8	NDCBE-A	NP_001035049.1	Human	
	NDCBE-B	AAC82380.1	Human	
	NDCBE-C	NP_001245330.1	Human	
	NDCBE-D	ABJ91577	Human	
	NDCBE-E	BAA34459.1	Human	
	THYMU3021755	Nuc# AK128321.1	Human	
SLC4A10	NBCn2-A	NP_071341.2	Human	
	NBCn2-B	NP_001171486.1	Human	
	NBCn2-C	NP_001229308.1	Mouse	
	NBCn2-D	NP_001229307.1	Mouse	
	NBCn2-E	AFP48940.1	Rat	
	NBCn2-F	AFP48941.2	Rat	
	NBCn2-G	AFP48942.1	Rat	
	NBCn2-H	AFP48943.2	Rat	
	NBCn2-I	AFQ60533.1	Mouse	
	NBCn2-J	AFN27376.1	Mouse	
	NBCn2-K	AHG54969.1	Rat	
	NBCn2-L	AHG54968.1	Rat	
	NBCn2-M	AHG54967.1	Rat	
	NBCn2-N	AHG54966.1	Rat	
	rb3NCBE	AY579373.1	Rat	
	rb7NCBE	AAS89266.1	Rat	
SLC4A9	AE4a	NM_031467	Human	
	AE4a	BAC10662.1	Rat	
	AE4a	AB038263	Rabbit	
	AE4b	AB038264	Rabbit	
